# Is traumatic brain injury a risk factor for neurodegeneration? A meta-analysis of population-based studies

**DOI:** 10.1186/s12883-018-1187-0

**Published:** 2018-11-05

**Authors:** Chi-Hsien Huang, Chi-Wei Lin, Yi-Che Lee, Chih-Yuan Huang, Ru-Yi Huang, Yi-Cheng Tai, Kuo-Wei Wang, San-Nan Yang, Yuan-Ting Sun, Hao-kuang Wang

**Affiliations:** 1 Department of Family Medicine, E-Da Hospital, I-Shou University, Kaohsiung, Taiwan; 2 School of Medicine for International Students, I-Shou University, Kaohsiung, Taiwan; 3 Department of Nephrology, E-Da Hospital, I-Shou University, Kaohsiung, Taiwan; 4 Neurosurgical Service, Department of Surgery, National Cheng Kung University Hospital, Tainan, Taiwan; 5 Department of Neurology, E-Da Hospital, I-Shou University, Kaohsiung, Taiwan; 6 Department of Neurosurgery, E-Da Hospital, I-Shou University, No.1, Yida Road, Jiaosu Village, Yanchao District, Kaohsiung City, 82445 Taiwan; 7 Department of Neurology, National Cheng Kung University Hospital, College of Medicine, National Cheng Kung University, Tainan, Taiwan

**Keywords:** TBI, Dementia, Neurodegeneration, Meta-analysis

## Abstract

**Background:**

To determine the association of prior traumatic brain injury (TBI) with subsequent diagnosis of neurodegeneration disease.

**Methods:**

All studies from 1980 to 2016 reporting TBI as a risk factor for diagnoses of interest were identified by searching PubMed, Embase, study references, and review articles. The data and study design were assessed by 2 investigators independently. A meta-analysis was performed by RevMan 5.3.

**Results:**

There were 18 studies comprising 3,263,207 patients. Meta-analysis revealed a significant association of prior TBI with subsequent dementia. The pooled odds ratio (OR) for TBI on development of dementia, FTD and TDP-43 associated disease were 1.93 (95% CI 1.47–2.55, *p* < 0.001), 4.44 (95% CI 3.86–5.10, p < 0.001), and 2.97 (95% CI 1.35–6.53, p < 0.001). However, analyses of individual diagnoses found no evidence that the risk of Alzheimer’s disease, and Parkinson’s disease in individuals with previous TBI compared to those without TBI.

**Conclusions:**

History of TBI is not associated with the development of subsequent neurodegeneration disease. Care must be taken in extrapolating from these results because no suitable criteria define post TBI neurodegenerative processes. Therefore, further research in this area is needed to confirm these questions and uncover the link between TBI and neurodegeneration disease.

## Introduction

Traumatic brain injury (TBI) occurs when an external force injures the brain. Motor vehicle accidents cause most TBIs in young adults, while falls are the leading cause of TBIs in people over 65 years of age [1]. Owing to the increasing use of motor vehicles in developed countries, the incidence of TBI is rapidly growing. TBI is a major cause of death and disabilities—especially in children and young adults—that result in high societal costs [1, 2]. Men sustain TBIs more frequently than women do. These observations suggest that TBIs result in major health and socioeconomic problems throughout the world. Indeed, the World Health Organization has reported that traffic accidents are the third highest contributor to the global burden of disease and injury [1, 2].

Neurological damage occurs not only at the moment of impact (primary injury) in a TBI, but also it further develops overtime post-impact. Several processes, such as neurotransmitter release, free-radical generation, calcium-mediated damage, gene activation, mitochondrial dysfunction, and inflammatory responses, have been investigated in studies of the secondary injuries that are associated with TBI [3–5]. These mechanisms might occur continuously over patients’ lifetimes. Interestingly, a history of TBI has been reported to increase the incidence of Alzheimer disease (AD) [6] and other neurodegenerative conditions, including Parkinson’s disease (PD) [7], amyotrophic lateral sclerosis (ALS) [8], and frontotemporal dementia (FTD) [5]. However, other studies have yielded contradicting results [9–13].

The goal of our study was to determine whether TBI was associated with an increased risk of neurodegeneration by conducting a systematic review of cohort studies of patients with TBI. In addition, we examined which neurodegenerative process occurred most often.

## Methods

### Data sources

We conducted systematic literature searches of the association between neurodegeneration and TBI in the Medical Literature Analysis and Retrieval System Online (MEDLINE®)/PubMed (US National Library of Medicine, National Institutes of Health, Bethesda, MD; http://www.ncbi.nlm.nih.gov/pubmed) database and Excerpta Medica Database (EMBASE®, Elsevier, Amsterdam, Netherlands; http://www.elsevier.com/solutions/embase-biomedical-research). We used the following keywords to generate a list of potentially useful studies: ([traumatic brain injury] OR [head injury] OR [brain injury] OR [TBI]) AND ([neurodegeneration] OR [cognitive dysfunction] OR [dementia] OR [alzheimer’s disease] OR [AD] OR [parkinson’s disease] OR [parkinsonism] OR [frontotemporal dementia] OR [Amyotrophic Lateral Sclerosis]) [14–16]. The search was performed through December 2016. The reference lists in the selected studies, as well as the list of studies included in earlier meta-analyses on similar topics, were reviewed for additional references. This review considered observational studies, and case series.

### Inclusion criteria

We included published articles on the risk of neurodegeneration, including cognitive dysfunction, dementia, AD, PD, FTD, and ALS, among individuals with TBIs compared with the risk of neurodegeneration in individuals in a nonbrain-injured population-based control group. A risk estimate was calculated with the data provided in the article. TBI was not limited in accordance with its severity.

### Exclusion criteria

Studies were excluded if (1) they were reviews, case reports, or case series; (2) they only consisted of a follow-up study of a cohort of patients with brain injury with no comparison group; (3) there was a non-population-based control group (i.e., a patient control group); or (4) insufficient information was available to allow for calculations of risk estimates [14–16].

### Study selection

The outcomes recorded were neurodegeneration, dementia, PD, and transactive response DNA-binding protein of 43 kDa (TDP-43) aggregation-associated disease. ALS and FTD, which are closely related conditions with overlapping clinical, pathological, radiological, and genetic characteristics, are characterized by TDP-43 aggregation [17]. Therefore, we defined ALS and FTD as TDP-43-associated diseases. Two authors (H.K. Wang or Y.C. Tai) examined the titles and abstracts of the studies found in the systematic literature search. The entire articles of potentially eligible studies were assessed to determine if the studies met the criteria. The study selection process was performed in accordance with the Preferred Reporting Items for Systematic Reviews and Meta-Analyses (PRISMA) guidelines and documented with a PRISMA flow diagram.

### Data analysis

The effects of TBI on the neurodegeneration outcomes were assessed with a random effects model because the designs and patient populations of the observational studies were expected to be heterogeneous. The Odds Ratio (OR) and 95% confidence intervals (CIs) were calculated. Statistical heterogeneity was examined with chi-square and I-squared (I^2^) tests of heterogeneity. The data were analyzed with Review Manager (RevMan) Version 5.3 (Copenhagen: The Nordic Cochrane Centre, The Cochrane Collaboration, 2014).

## Results

### The systematic search

Using the search terms, our literature and reference list searches yielded 1317 references. After examination of the abstracts and, when indicated, the full texts of the articles, 68 studies were considered potentially relevant [18–37] . Finally, 18 studies met our inclusion criteria and were included in the meta-analysis. The characteristics of the included trials are listed in Table 1. Methodologic qualities of are shown in Table 2.

**Table 1:** Individual for all included studies

Ref	Author, year	Type	TBI+ Neurodegeneration +	TBI+ Neurodegeneration -	TBI- Neurodegeneration +	TBI- Neurodegeneration -
20	Mehta KM, et al. 1999	Dementia (APOE)	11	788	118	5728
21	Luukinen H, et al. 2005		5	3	29	115
22	Sundström A, 2007		25	46	156	316
23	Luukinen H, et al. 2008		11	17	14	92
25	Wang HK, 2012	Dementia	1196	43,729	3499	221,186
26	Gardner RC, et al. 2014		4361	47,438	6610	108,891
27	Nordström P, et al.2014		108	45,141	458	765,915
28	Abner EL, et al. 2014		8	158	31	452
29	Rasmusson DX, et al. 1995	AD	20	1	48	33
30	Nemetz PN, et al. 1999		31	1252	957	2783
31	Barnes DE, et al. 2014		12	1217	1098	186,437
32	Spangenberg S, et al. 2009	Parkinson	87	73,232	8769	2,799,434
33	Lee PC, et al. 2012		42	50	315	704
34	Taylor KM, et al. 2015		69	24	310	206
35	Gardner RC, et al. 2015		891	51,502	1247	112,159
36	Kalkonde YV, 2012	FTD (TDP-43)	8	17	55	474
5	Wang HK, et al. 2015		363	24,222	413	122,512
37	Chen H, et al. 2007	ALS (TDP-43)	24	42	85	213

**Table 2:** Consensus ACROBAT-NRSI judgments between two reviewers by domain of bias

Component Study	Domain							Overall RoB Bias
	Bias Due to Judgment Confounding	Bias in Selection of Participants	Bias in Measurement of Interventions	Bias Due to Departures from Intended Interventions	Bias in Measurement of Outcomes	Bias in Selection of Reported Results	Bias Due to Missing Data	
Mehta KM, et al. [20]	moderate	moderate	low	low	low	low	low	moderate
Luukinen H, et al. [21]	low	low	low	low	low	low	low	low
Sundström A, [22]	low	low	low	low	low	low	low	low
Luukinen H, et al. [23]	low	low	low	low	low	low	low	low
Wang HK, [25]	low	moderate	low	low	moderate	low	low	low
Gardner RC, et al. [26]	low	moderate	low	low	moderate	low	low	low
Nordström P, et al. [27]	low	moderate	low	low	moderate	low	low	low
Abner EL, et al. [28]	low	moderate	low	low	moderate	low	low	low
Rasmusson DX, et al. [29]	low	low	low	low	low	low	low	low
Nemetz PN, et al. [30]	low	moderate	low	low	moderate	low	low	low
Barnes DE, et al. [31]	low	moderate	low	low	moderate	low	low	low
Spangenberg S, et al. [32]	low	low	low	low	low	low	low	low
Lee PC, et al. [33]	low	low	low	low	low	low	low	low
Taylor KM, et al. [34]	low	low	low	low	low	low	low	low
Gardner RC, et al. [35]	low	moderate	low	low	moderate	low	low	low
Kalkonde YV, [36]	low	low	low	low	low	low	low	low
Wang HK, et al. [5]	low	moderate	low	low	moderate	low	low	low
Chen H, et al. [37]	low	low	low	low	low	low	low	low

### The subgroup meta-analyses

**Fig. 1:**
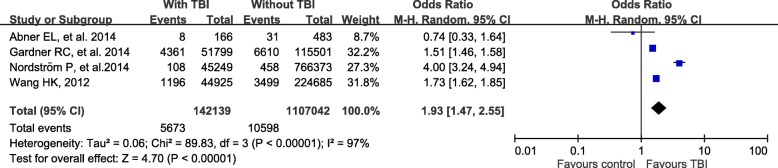
Individual and pooled odds ratios for dementia

**Fig. 2:**
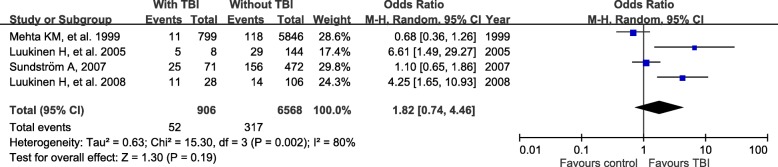
Individual and pooled odds ratios for dementia with APOE

**Fig. 3:**
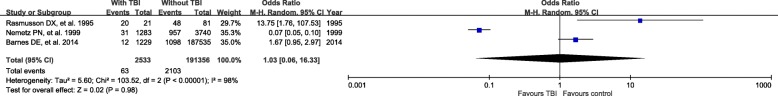
Individual and pooled odds ratios for Alzheimer’s Disease (AD)

**Fig. 4:**
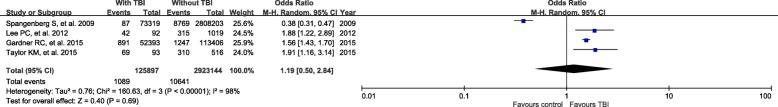
Individual and pooled odds ratios for Parkinson’s Disease (PD)

**Fig. 5:**
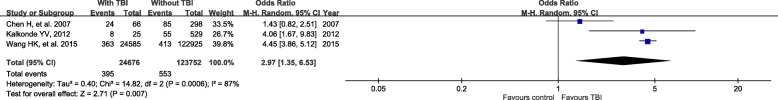
Individual and pooled odds ratios for transactive response DNA-binding protein of 43 kDa (TDP-43)

**Fig. 6:**
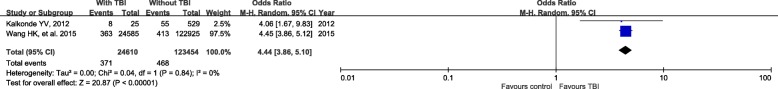
Individual and pooled odds ratios for frontotemporal dementia (FTD)

There was an association between TBI and subsequent dementia (pooled OR = 1.93, 95% CI = 1.47–2.55) (Fig. 1). However, this association does not concur with the apolipoprotein E (APOE) genotype (pooled OR = 1.82, 95% CI = 0.74–4.46) (Fig. 2). The studies associated with AD yielded a pooled OR of 1.03 (95% CI = 0.06–16.33) with heterogeneity (I^2^ = 98%, *P* < 0.001) (Fig. 3). The analysis of the risk of TBI that was associated with PD yielded a pooled OR of 1.19 (95% CI = 0.50–2.84) with heterogeneity (I^2^ = 98%, P < 0.001) (Fig. 4). The analysis of the risk associated with TDP-43 and FTD yielded a pooled OR of 2.97 (95% CI = 1.35–6.53) and 4.44 (95% CI = 3.86–5.10) (Figs. 5 and 6).

## Discussion

Concerns about the relationship between TBI and neurodegeneration have long existed. Our principal finding was that TBI is a potential risk factor for subsequent dementia (1.93, 95% CI = 1.47–2.55), TDP-43 (2.97, 95% CI = 1.35–6.53) and FTD (4.44, 95% CI = 3.86–5.10). However, when we analyzed the associations of the incidences of AD, PD, or dementia with APOE genotype, we found that TBI was not a risk.

A common factor between TBI and different neurodegenerative disorders is the abnormal aggregation, accumulation, and/or disposition of proteins in the brain. Patients with TBI have beta-amyloid deposition in their brains, and the pattern of deposition is similar to that observed in patients with AD. Amyloid precursor protein, β-Amyloid precursor protein-cleaving enzyme-1, and presenilin-1, which is a γ-secretase complex protein, serve as sources of amyloid-β peptide deposition after TBI [5, 29–31].

TBI may also exacerbate nigrostriatal dopaminergic degeneration by modulating PD-associated genes. These results suggest that α-synuclein is a pathological link between the chronic effects of TBI and PD symptoms [32–34].

Another mechanism that might link TBI to neurodegeneration is the accumulation of TDP-43 in patients with FTD and ALS [5, 24, 37]. Patients with chronic traumatic encephalopathy (CTE) exhibit widespread TDP-43 proteinopathy in multiple areas of the brain [38]. Some patients also had TDP-43 protein in their spinal cords, and these patients developed a progressive motor neuron disease several years before their deaths [38, 39]. These findings suggest that TDP-43-associated neurodegeneration and head trauma are connected.

The mechanisms underlying the association of TBI and the incidence of neurodegeneration are exceedingly complex. In fact, in TBI, multiple pathologies occur simultaneously. However, systematic analyses of multiple pathologies in individual cases in a large TBI and control population have not been conducted [9–13, 39]. Therefore, a significant association of AD, PD, and APOE-associated dementia was not observed, and no evidence that head trauma was a risk factor for patients with these disorders was found.

Another noteworthy finding of our study was that AD is not the most common form of neurodegeneration. Several studies have reported that TBI does not affect the development of AD. Whether a single occurrence of moderate-to-severe TBI triggers the development of neurodegeneration remains somewhat controversial [9–13]. In addition, brain injury has been shown to lead to the development of non-AD dementias. However, accurate clinical diagnostic criteria for neurodegeneration that results from TBI do not exist. Therefore, the true risk of neurodegeneration following TBI is hard to define.

Examinations of patients with CTE may help resolve this problem. The symptoms of CTE, which include behavioral disturbances, cognitive dysfunction, and/or motor-related symptoms, generally begin 8–10 years after repetitive mild TBIs are experienced [40–42]. However, the clinical diagnostic criteria for traumatic encephalopathy syndrome have only recently been reported. CTE can be seen after a single moderate or severe TBI while traumatic encephalopathy syndrome exhibits progressive deterioration over time. Therefore, clinical judgment must be used to determine whether the amount of progression is greater than what is expected for the age and comorbidities of the patient [39–42]. Multiple neurodegeneration processes have been used to describe the behavioral disturbances and cognitive dysfunction in patients following TBI owing to the lack of suitable diagnoses. This diagnosis can now be used to define those symptoms.

A major strength of this meta-analysis was the inclusion of a variety of different neurodegenerative outcomes rather than a single diagnosis. The literature search was comprehensive because it focused on diagnoses rather than self-reported symptoms, which resulted in a rigorous examination of the topic.

However, several limitations of this meta-analysis warrant consideration. First, the initial severities of the TBIs varied within and across studies. Since our meta-analyses examined only published data, some important parameters, such as sex, alcohol consumption, and comorbidities were missing. Therefore, we were not able to control for these potential confounding factors. Patients with severe TBI had loss of high cortical function and presented in a vegetative state. In our studies, we included only patients who sought treatment for TBI and neurodegeneration disease, but not important parameters indicating clinical severity and imaging information on TBI. Therefore, it is hard to understand the relationship between the severity of TBI and neurodegeneration disease.

Second, we were not able to examine the effects of the locations of the TBIs in this analysis. Some investigators have proposed that temporal and frontal lobe lesions are more likely to be associated with an increased risk of later dementia compared with lesions in other brain regions [5]. In addition, preinjury intelligence and education level are also associated with TBI and dementia. However, the effects of these factors in this study were difficult to determine.

Another limitation was that none of the studies included in this review provided information on genetics [8]. The estimated effect of the apolipoprotein E (*APOE*) gene 4 on functional outcome might be greatly confounded by such factors, which could thus influence the validity of any meta-analysis that uses unadjusted results. Therefore, future studies on the relationship between *APOE 4* expression and the functional outcome of TBI should consider these important confounding factors. The age may also have an effect on the patients with neurodegeneration diseases. The risk between early-onset and late-onset neurodegeneration diseases maybe different. The patient with young onset neurodegeneration diseases more likely has a genetic or metabolic disease. However, neurodegeneration diseases are far more common in geriatric population, and researches in neurodegeneration disease are focused mainly on old persons. Therefore, it is hard to understand the association between age and neurodegeneration diseases in our study.

## Conclusion

Patients with TBI frequently exhibit neurodegeneration. The symptoms of these neurodegenerative processes include behavioral disturbances, cognitive dysfunction, and/or motor-related symptoms. TBI is a potential risk factor only for subsequent dementia, TDP-43, and FTD, but not for AD, PD, or APOE-associated neurodegeneration. The epidemiology and pathology of this association have been difficult to establish. No suitable criteria define these neurodegenerative processes. However, the diagnosis of CTE may help to resolve this problem. Although the evidence suggested that TBI was a risk factor for neurodegeneration, there is limited information on the type, frequency, or amount of trauma that was necessary to induce the neurodegenerative processes. Therefore, further studies in this area are necessary to answer these questions and determine whether the proper management of TBI is effective in reducing the incidence of neurodegeneration.

## Abbreviations

AD: Alzheimer disease
ALS: amyotrophic lateral sclerosis
APOE: apolipoprotein E
FTD: frontotemporal dementia (FTD)
PD: Parkinson’s disease
TBI: traumatic brain injury
TDP-43: transactive response DNA-binding protein of 43 kDa
